# Risk factor assessments of temporomandibular disorders via machine learning

**DOI:** 10.1038/s41598-021-98837-5

**Published:** 2021-10-05

**Authors:** Kwang-Sig Lee, Nayansi Jha, Yoon-Ji Kim

**Affiliations:** 1 AI Center, Korea University College of Medicine, Seoul, Korea; 2 Department of Orthodontics, Asan Medical Center, University of Ulsan College of Medicine, 88 Olympic-ro 43-gil, Songpa-gu, Seoul, 05505 Korea

**Keywords:** Medical research, Pathogenesis, Risk factors

## Abstract

This study aimed to use artificial intelligence to determine whether biological and psychosocial factors, such as stress, socioeconomic status, and working conditions, were major risk factors for temporomandibular disorders (TMDs). Data were retrieved from the fourth Korea National Health and Nutritional Examination Survey (2009), with information concerning 4744 participants’ TMDs, demographic factors, socioeconomic status, working conditions, and health-related determinants. Based on variable importance observed from the random forest, the top 20 determinants of self-reported TMDs were body mass index (BMI), household income (monthly), sleep (daily), obesity (subjective), health (subjective), working conditions (control, hygiene, respect, risks, and workload), occupation, education, region (metropolitan), residence type (apartment), stress, smoking status, marital status, and sex. The top 20 determinants of temporomandibular disorders determined via a doctor’s diagnosis were BMI, age, household income (monthly), sleep (daily), obesity (subjective), working conditions (control, hygiene, risks, and workload), household income (subjective), subjective health, education, smoking status, residence type (apartment), region (metropolitan), sex, marital status, and allergic rhinitis. This study supports the hypothesis, highlighting the importance of obesity, general health, stress, socioeconomic status, and working conditions in the management of TMDs.

## Introduction

Temporomandibular disorders (TMDs) are common painful craniofacial conditions involving the temporomandibular joint and the masticatory muscles^1–3^. They are the second most common musculoskeletal condition after back pain^4^. TMD symptoms include jaw pain, mouth opening limitations, sound in the preauricular area, and mouth opening deviation^3^. These disorders can be classified as pain-related (which include masticatory muscle disorders and inflammation of the temporomandibular joint (TMJ) capsule) and intra-articular disorders (which include TMJ disc displacement and osteoarthritis)^5^. The rate of occurrence of signs and symptoms associated with TMDs is reported to range from 1.8 to 33.4% across different populations^6^. Its annual associated financial costs amount to > 100.0 billion dollars in the United States^7^. In South Korea, the number of patients diagnosed with TMDs has increased by approximately 17.1%, and the related national health insurance cost has increased by approximately 47.3% between 2015 and 2019^8^.

The etiology of TMDs is considered multifactorial, with biological and psychosocial factors contributing either independently, or as interrelated factors^9^. Clinical factors directly related to the jaw function include trauma, parafunction, unstable occlusion, functional overloading^10–16^ and psychosocial factors—such as mental stress, socioeconomic status, and work environments; both categories have been reported as being predisposed to promote TMD onset and persistence^17–22^. In addition, comorbid conditions—such as cardiovascular diseases, osteoarthritis, tinnitus, sinusitis, allergic rhinitis, and thyroid disorders—have been found to contribute to the prognosis of TMDs^23–25^.

Despite the various etiological factors that have been identified, little is known about the independent or interrelated roles of each of these factors and their relative contributions to the development of TMDs. Therefore, this study aimed to identify the contributing biological and psychosocial factors and their relative importance as risk factors for the development of TMDs. This study uses artificial intelligence methodologies on a nationwide sample of 4744 participants so as to classify the etiological factors in order of importance—including demographic factors, socioeconomic status, mental stress, working environment, health-related variables, and comorbid conditions.

## Methods

### Study population and sampling

Data were obtained from the fourth Korea National Health and Nutritional Examination Survey, that is, KNHANES IV-3 2009^26^. KNHANES is a nationwide annual cross-sectional survey conducted by the Division of Chronic Disease Surveillance of the Korea Disease Control and Prevention Agency. The survey collects information from approximately 10,000 nationally-representative and noninstitutionalized civilians in Korea regarding their socioeconomic status, health-related behaviors, quality of life, healthcare utilization, anthropometric parameters, biochemical and clinical profiles for noncommunicable diseases, and dietary intake. The data are de-identified and publicly available upon request. The requirement for ethical approval from the institutional review board of the Asan Medical Center was waived (waiver number: 2020-0362). All methods were conducted in accordance with relevant guidelines and regulations.

The final sample consisted of 4744 participants aged ≥ 19 years with information about TMDs. According to the KNHANES IV 2009 survey, participants who responded “yes” to the question “Have you had symptoms related to a TMD such as jaw pain, joint sound, and/or mouth opening limitations?” were considered as self-reported TMD (r-TMD) patients. Those who responded “yes” to the question “Have you been told by a doctor that you have a TMD?” were considered as TMD patients as diagnosed by a doctor (d-TMD). Those who responded “no” to these questions were categorized in a control group of those without TMDs. Information about both r-TMD and d-TMD was available only in the KNHANES IV-3 survey conducted in 2009.

In total, 37 independent variables were analyzed from the survey. These include: (1) demographic factors, (2) socioeconomic status, (3) mental stress/working environment, (4) biological variables, and (5) comorbidities. The full list of variables included is shown in Table 1.

**Table 1:** Relevant variables from the Fourth Korea National Health and Nutritional Examination Survey.

Category	Variable
Demographic	Age
	Sex
	Region: metropolitan
	Region: rural
	Residence type: apartment
Socioeconomic	Household income—monthly (USD)
	Household income—subjective
	Education
	Occupation
	Marital status
	Health insurance
Mental stress/working environment	Stress
	Suicidal ideation
	Working environment—hygienic environment
	Working environment—harmful or hazardous work
	Working environment—workload
	Working environment—decision making authority
	Working environment—respected by colleagues
Biological variables	Smoking status
	Subjective health
	Obesity—subjective
	Drinking
	Body mass index
	Sleep—daily (hours)
Comorbidities	Hypertensive disorders
	Rheumatoid arthritis
	Osteoarthritis
	Osteoporosis
	Lumbago
	Sinusitis
	Allergic rhinitis
	Mental depression
	Atopic dermatitis
	Diabetes mellitus
	Thyroid disorders
	Otitis media
	Gastric/duodenal ulcer

### Analysis

Six artificial intelligence approaches were used for identifying the factors associated with TMDs, and the accuracy of each model was compared: logistic regression, decision trees, naïve Bayes, random forest, support vector machines, and an artificial neural network. The following hyper parameters were used for these methods: GINI was considered as the impurity measure of the decision tree, 1000 was regarded as the number of decision trees in the random forest, radial basis functioning was used as the kernel of the support vector machine, and 10-10 was the sizes utilized for two hidden layers and quasi-Newton (lbfgs) as weight optimization in the artificial neural network (https://scikit-learn.org/stable/index.html). The data on 4744 participants were divided into training and validation sets at a 75:25 ratio. The models were trained based on the training set with data from 3558 participants and then validated using the validation set using data from 1186 participants. This validation set was not involved in the training (or learning) of machine learning approaches but was designed to only validate (or evaluate) their performance. Accuracy—defined as the rate of correct predictions from the data from 1186 participants—was used as a criterion for validating the trained models. Variable importance—an accuracy (or mean-impurity) gap between a complete model and a model excluding a certain variable^27^—was analyzed from the random forest model to test the study hypothesis, which is to assess the impact of each variable in predicting the presence of TMDs. For example, let us assume that the variable importance of “household income” is 0.10; the accuracy of the random forest will decrease by 0.10 if household income is excluded from the model. In other words, the variable importance of a certain variable measures the degree of its contribution to the performance of the model. From the independent variables included, the top 20 variables (in order of importance) were considered as risk factors for TMDs. Python 3.52 (Centrum voor Wiskunde en Informatica, Amsterdam, Netherlands) was used for statistical analysis in September 2020. For the logistic regression, odds ratios were calculated, and a P-value of < 0.05 was considered as being statistically significant.

## Results

Descriptive statistics for participants’ categorical and continuous variables are shown in Appendix Table 1 and Table 2, respectively. Among the 4744 participants (2479 males and 2265 females, with a median age of 45 years), 101 (2.1%), and 68 (1.4%) had a r-TMD and a d-TMD, respectively. Forty-three individuals (0.9%) were included as both r-TMD and d-TMD. Of all respondents, 42% reported living in a metropolitan area, while 28.4% lived in a rural area (Appendix Table 1). Regarding the level of education, 66.8% had at least a high school education (Appendix Table 1). The median monthly household income, body mass index (BMI), and number of hours of sleep were $2500, 23.47, and 7 h, respectively (Table 2).

**Table 2:** Descriptive statistics: continuous variables.

Variable	Min	Q1	Q2	Q3	Max
Age	19.00	34.00	45.00	56.00	80.00
Household income—monthly (USD)	17.00	1500.00	2500.00	4000.00	9000.00
Body mass index	0.00	21.31	23.47	25.73	40.54
Sleep—daily (h)	1.00	6.00	7.00	8.00	13.00

Table 3 shows the performance of the machine learning models that were tested. The greatest mean accuracy was observed via methods employing logistic regression, random forest, support vector machines, and artificial neural networks for both r-TMD and d-TMD; the greatest area under the receiver-operating-characteristic curve (AUC) was observed via the use of an artificial neural network and logistic regression for r-TMD and d-TMD, respectively (Table 3).

**Table 3:** Model performance.

Model	Run 1		Run 2		Mean	
	Accuracy	AUC	Accuracy	AUC	Accuracy	AUC
**TMD (self-reported)**						
Logistic Regression	0.9823	0.64	0.9755	0.66	0.9789	0.65
Decision Tree	0.9823	0.49	0.9410	0.48	0.9617	0.49
Naïve Bayes	0.8423	0.63	0.8255	0.62	0.8339	0.63
Random Forest	0.9823	0.63	0.9755	0.65	0.9789	0.64
Support Vector Machine	0.9823	0.43	0.9755	0.49	0.9789	0.46
Artificial Neural Network	0.9823	0.69	0.9755	0.66	0.9789	0.68
**TMD (diagnosed by doctor)**						
Logistic Regression	0.9874	0.69	0.9806	0.71	0.9840	0.70
Decision Tree	0.9688	0.56	0.9680	0.51	0.9684	0.54
Naïve Bayes	0.8558	0.64	0.6804	0.68	0.7681	0.66
Random Forest	0.9874	0.57	0.9806	0.62	0.9840	0.60
Support Vector Machine	0.9874	0.60	0.9806	0.55	0.9840	0.58
Artificial Neural Network	0.9874	0.60	0.9806	0.74	0.9840	0.67
Under-sampling 101:404*						
TMD (self-reported)						
Logistic Regression	0.8268	0.70	0.8976	0.80	0.8622	0.75
Decision Tree	0.6929	0.54	0.8661	0.78	0.7795	0.66
Naïve Bayes	0.4173	0.59	0.8504	0.79	0.6339	0.69
Random Forest	0.8031	0.71	0.9134	0.76	0.8583	0.74
Support Vector Machine	0.8346	0.43	0.8189	0.50	0.8268	0.46
Artificial Neural Network	0.8425	0.68	0.8268	0.71	0.8346	0.69
Under-sampling 101:404*						
TMD (diagnosed by doctor)						
Logistic Regression	0.8898	0.62	0.9055	0.61	0.8976	0.62
Decision Tree	0.8189	0.59	0.8425	0.47	0.8307	0.53
Naïve Bayes	0.4409	0.71	0.3307	0.67	0.3858	0.69
Random Forest	0.8976	0.61	0.9055	0.67	0.9016	0.64
Support Vector Machine	0.8976	0.54	0.9055	0.63	0.9016	0.58
Artificial Neural Network	0.8976	0.66	0.9055	0.74	0.9016	0.70
Under-sampling 101:202*						
TMD (self-reported)						
Logistic Regression	0.7500	0.76	0.6711	0.60	0.7105	0.68
Decision Tree	0.7105	0.67	0.5526	0.48	0.6316	0.58
Naïve Bayes	0.6579	0.68	0.5789	0.60	0.6184	0.64
Random Forest	0.7368	0.71	0.6053	0.60	0.6711	0.66
Support Vector Machine	0.7368	0.65	0.6711	0.63	0.7039	0.64
Artificial Neural Network	0.7632	0.79	0.6316	0.65	0.6974	0.72
Under-sampling 101:909*						
TMD (Self reported)						
Logistic Regression	0.9130	0.65	0.9091	0.64	0.9111	0.65
Decision Tree	0.8142	0.51	0.8142	0.55	0.8142	0.53
Naïve Bayes	0.7075	0.65	0.7233	0.56	0.7154	0.61
Random Forest	0.9051	0.66	0.9091	0.64	0.9071	0.65
Support Vector Machine	0.9051	0.61	0.9091	0.63	0.9071	0.62
Artificial Neural Network	0.9130	0.61	0.9091	0.65	0.9111	0.63

*TMD* temporomandibular disorders, *AUC* area under the curve.

*404, 202 or 909 observations were randomly sampled without replacement from 4643 observations with TMD Self-Reported “No” (The proportion of observations with TMD Self-Reported “Yes” became 20%, 30% or 10%).

The proportions of r-TMD and d-TMD are minimal; that is, 2.1% and 1.4%, respectively. This caused a class-imbalance problem. The machine learning approach was trained to classify all observations as r-TMD “No” or d-TMD “No”. This led to a high degree of accuracy but a low level of AUC. A possible solution is the use of under-sampling, as reported in Table 3: 404, 202, and 909 observations were randomly sampled without replacement from 4643 observations with r-TMD “No” (the proportions of observations with r-TMD “Yes” became 20%, 30%, and 10%, respectively). These approaches are referred to as under-sampling 101:404, 101:202, and 101:909, respectively. Although the accuracy of logistic regression and the random forest methodology decreased to 0.86, their corresponding AUC values increased to 0.75 and 0.74 (respectively) in the case of under-sampling 101:404 (r-TMD). The AUC values did not improve in the other cases of under-sampling (see Table 3).

**Figure 1:**
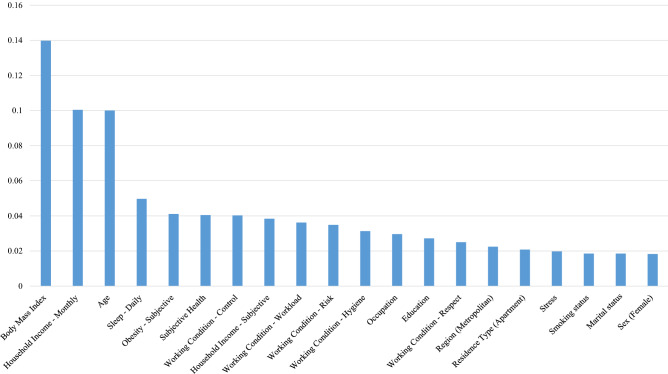
Based on variable importance from the random forest, the top 20 determinants of self-reported TMDs were body mass index, household income (monthly), age, sleep (daily), obesity (subjective), subjective health, working conditions (control, hygiene, respect, risks, and workload), subjective household income, occupation, education, region (metropolitan), residence type (apartment), stress, smoking status, marital status, and sex.

**Figure 2:**
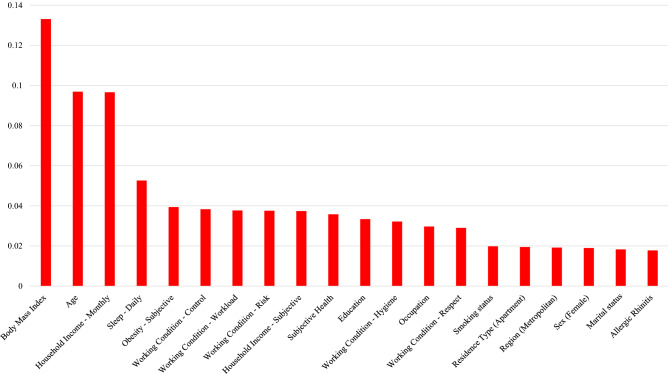
The results of variable importance from the random forest, the top 20 determinants of TMDs as diagnosed by a doctor: body mass index, age, household income (monthly), sleep (daily), obesity (subjective), working condition (control, hygiene, respect, risks, and workload), household income (subjective), subjective health, education, occupation, smoking status, residence type (apartment), region (metropolitan), sex, marital status, and allergic rhinitis.

**Figure 3:**
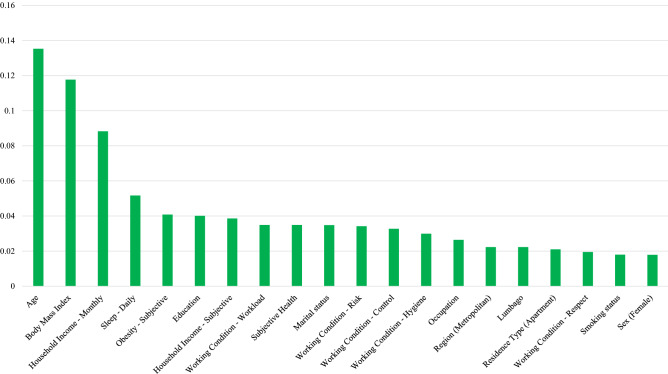
Based on variable importance from the random forest with under-sampling (101:404), the top 20 determinants of self-reported TMDs are age, body mass index, household income (monthly), sleep (daily), obesity (subjective), education, household income (subjective), working conditions (control, hygiene, respect, risks, and workload), subjective health, marital status, occupation, region (metropolitan), lumbago, residence type (apartment), smoking status, and sex.

Based on variable importance determined from the random forest, the top 20 determinants of r-TMD, d-TMD, and r-TMD (under-sampling 101:404) are shown in Table 4 and in Figs. 1, 2 and 3.

**Table 4:** Random forest variable importance.

Variable	Value	Rank
**TMD (self-reported)**		
Body mass index	0.1398	1
Household income—monthly	0.1004	2
Age	0.1000	3
Sleep—daily	0.0497	4
Obesity—subjective	0.0411	5
Subjective health	0.0405	6
Working condition—control	0.0403	7
Household income—subjective	0.0384	8
Working condition—workload	0.0362	9
Working condition—risk	0.0349	10
Working condition—hygiene	0.0313	11
Occupation	0.0296	12
Education	0.0272	13
Working condition—respect	0.0250	14
Region (metropolitan)	0.0225	15
Residence type (apartment)	0.0208	16
Stress	0.0198	17
Smoking status	0.0185	18
Marital status	0.0185	19
Sex (female)	0.0183	20
Lumbago	0.0174	21
Region (rural)	0.0172	22
Suicidal ideation	0.0170	23
Allergic rhinitis	0.0150	24
Otitis media	0.0128	25
Thyroid disorders	0.0105	26
Sinusitis	0.0088	27
Rheumatoid arthritis	0.0079	28
Gastric/duodenal ulcer	0.0066	29
Drinking	0.0064	30
Mental depression	0.0059	31
Osteoarthritis	0.0052	32
Hypertensive disorders	0.0047	33
Atopic dermatitis	0.0039	34
Diabetes mellitus	0.0029	35
Osteoporosis	0.0027	36
Health insurance	0.0022	37
**TMD (diagnosed by doctor)**		
Body mass index	0.1331	1
Age	0.0969	2
Household income—monthly	0.0966	3
Sleep—daily	0.0526	4
Obesity—subjective	0.0394	5
Working condition—control	0.0383	6
Working condition—workload	0.0377	7
Working condition—risk	0.0376	8
Household income—subjective	0.0374	9
Subjective health	0.0358	10
Education	0.0334	11
Working condition—hygiene	0.0322	12
Occupation	0.0297	13
Working condition—respect	0.0290	14
Smoking status	0.0198	15
Residence type (apartment)	0.0195	16
Region (metropolitan)	0.0192	17
Sex (female)	0.0190	18
Marital status	0.0183	19
Allergic rhinitis	0.0178	20
Stress	0.0178	21
Lumbago	0.0174	22
Suicidal ideation	0.0174	23
Region (rural)	0.0159	24
Otitis media	0.0157	25
Sinusitis	0.0122	26
Thyroid disorders	0.0107	27
Mental depression	0.0095	28
Gastric/duodenal ulcer	0.0076	29
Drinking	0.0064	30
Atopic dermatitis	0.0052	31
Hypertensive disorders	0.0051	32
Health insurance	0.0042	33
Diabetes mellitus	0.0037	34
Rheumatoid arthritis	0.0034	35
Osteoarthritis	0.0027	36
Osteoporosis	0.0020	37
Under-sampling 101:404* **TMD self-reported**		
Age	0.1353	1
Body mass index	0.1177	2
Household income—monthly	0.0883	3
Sleep—daily	0.0517	4
Obesity—subjective	0.0408	5
Education	0.0401	6
Household income—subjective	0.0386	7
Working condition—workload	0.0349	8
Subjective health	0.0349	9
Marital status	0.0348	10
Working condition—risk	0.0342	11
Working condition—control	0.0327	12
Working condition—hygiene	0.0299	13
Occupation	0.0264	14
Region (metropolitan)	0.0223	15
Lumbago	0.0223	16
Residence type (apartment)	0.0210	17
Working condition—respect	0.0195	18
Smoking status	0.0180	19
Sex (female)	0.0179	20
Stress	0.0168	21
Allergic rhinitis	0.0167	22
Suicidal ideation	0.0154	23
Region (rural)	0.0134	24
Otitis media	0.0131	25
Thyroid disorders	0.0103	26
Sinusitis	0.0083	27
Drinker	0.0080	28
Gastric/duodenal ulcer	0.0068	29
Rheumatoid arthritis	0.0066	30
Osteoarthritis	0.0066	31
Hypertensive disorders	0.0060	32
Diabetes mellitus	0.0031	33
Atopic dermatitis	0.0028	34
Health insurance	0.0027	35
Mental depression	0.0012	36
Osteoporosis	0.0009	37

*404 observations were randomly sampled without replacement from 4643 observations with TMD Self-Reported “No” (The proportion of observations with TMD Self-Reported “Yes” became 20%).

The top five variables for r-TMD, d-TMD, and r-TMD (under-sampling 101:404) were the same: BMI, household income, age, number of hours of sleep, and perceived obesity. Another major factor for the presence of TMDs was the individual’s work environment. The five variables associated with work environment were rated within the top 20 variables for r-TMD, d-TMD and r-TMD (under-sampling 101:404).

The odds ratio, as a result of the logistic regression, is shown in Appendix Table 2. A statistically significant association was observed for the following variables: age, marital status, health insurance type, working condition (respect), stress, suicidal ideation, and comorbidities of lumbago and depression.

## Discussion

To our knowledge, this study is the first attempt made in applying artificial intelligence methodology in identifying the etiologic factors of TMDs as based upon large-scale, nationwide survey data of 4744 patients. A predictive model was developed using 37 independent variables regarding demographic factors, socioeconomic status, stress, working conditions, biological factors, and comorbidities, and which can be used as a decision-support system in the diagnosis of TMDs. Additionally, we analyzed the association between each etiologic factor and the presence of TMDs using the random forest variable importance measures. The random forest is a group of many decision trees with a majority vote concerning the dependent variable. For example, in a random forest with 1000 decision trees used in this study, 1000 training sets were sampled with replacements, 1000 decision trees were trained with the 1000 training sets, and the 1000 decision trees took a majority vote on the dependent variable. This explains the high reliability and immense popularity of the random forest method^28^. Moreover, the method is free from the assumption that all the other variables remained constant, which is the case for statistical models used in most previous studies on the subject.

According to the literature review and the survey results, TMDs have significant associations with various psychological factors (such as anxiety, depression, and stress^29–31^), working conditions (including employment, occupation, working schedule, and working hours^32^), a high socioeconomic status (such as a high household income and a high degree of education^33^), and other chronic diseases (such as osteoarthritis, sinusitis, allergic rhinitis, mental depression, and thyroid disorders^25^). The findings of the literature are consistent with those identified via this study of the associations between TMDs and sleep, stress, employment/occupation, household income, and education. These variables were among the top 20 determinants of r-TMD as identified through our study. It needs to be noted, however, that the findings obtained via logistic regression are based on an unrealistic assumption of *ceteris paribus* (“the all the other variables remain constant”). For this reason, the results of logistic regression need to be considered as just supplementary information to the variable importance in terms of the random forest method.

Our results indicated that BMI is the most important determinant for the presence of TMDs; this was the case in both r-TMD and d-TMD. Self-perceived obesity was also ranked as fifth most important variable in both measures of TMDs. Obesity is a leading cause of disability and is associated with increased overall mortality^34^. It is recognized that adipose tissue plays a role in regulating inflammation in addition to storing energy^35^. Generally, increased adiposity is associated with the increased production of proinflammatory molecules, resulting in a proinflammatory state. Obesity has been designated as a risk factor for chronic musculoskeletal pain^36–38^. It is also strongly associated with osteoarthritis^39^ and rheumatoid arthritis^40, 41^. A recent study has confirmed the critical role played by adipokines (cytokines secreted by adipose tissues) in the pathophysiologic features of osteoarthritis, concluding that mechanical overload cannot completely explain the aggravation of knee osteoarthritis^39^. This, in turn, indicates the possible impact had by adipokines. We speculate that this systemic effect of increased inflammatory cytokines from the increased adiposity may be associated with the pathogenesis of TMDs.

Another explanation could be that elevated BMI is associated with a low socioeconomic status^42^, which is another risk factor for TMDs. Similarly, it has been reported that arthritis and joint symptoms are highly prevalent among those with poor general health, a high BMI, and a low socioeconomic status^43–45^. In contrast, Busija et al. reported that the association between BMI and arthritis is strong, but relatively independent of one’s age and socioeconomic status^46^.

Unlike arthritis, there are limited studies on the association between obesity and TMDs. Furthermore, for those studies that do exist, their results remain contentious. For example, a significant relationship between TMDs and obesity was observed via the use of univariate analysis. However, the association was not significant when the effects of sex, the presence of a migraine, nonspecific somatic symptoms, and obstructive sleep apnea syndrome were controlled for^47^. According to the research presented via the OPPERA studies—a large prospective and case–control investigation—BMI was not associated with TMDs^30^. Among adolescents, it has been concluded that obesity is not a risk factor for TMDs^48^.

This paper’s efforts improve upon previous research concerning the importance of working conditions in managing TMDs. A recent study used the working schedule (shift vs. daytime) and working hours (< 40 vs 40–48, 49–60, > 60) as two aspects of working conditions concerning the prediction of TMDs^32^. This research has introduced five dimensions of working conditions (hygiene, risk, workload, control, and respect), with their importance rankings being higher than 20: control (7th), workload (9th), risk (10th), hygiene (11th) and respect (14th), indicating the most important determinants of r-TMD. These findings suggest that preventive measures concerning these working conditions should be considered for central health policies.

This study has some limitations. First, we made use of survey data; therefore, the presence of TMDs is based on the self-reporting of participants. However, both outcomes of r-TMD and d-TMD indicated similar risk factors. Further studies using diagnostic criteria such as DC/TMD^5^, which include detailed questionnaires on the signs and symptoms of TMD and have additional subclassifications of pain related TMDs and intra-articular disorders, may be warranted; the artificial intelligence model could be fine-tuned using such additional data. Another limitation is that this research applied a cross-sectional design; hence, we were only able to observe the associations between risk factors and the presence of TMDs, and their causal effects could not be identified. Moreover, the prevalence of TMDs was relatively low in our sample compared with its worldwide prevalence. The prevalence of TMDs varies greatly depending on the diagnostic criteria and the target population^6^. Also, Asians have a lower prevalence of TMDs than whites and African Americans^20^. Due to the low prevalence of participants with TMDs, there was a class imbalance; therefore, down-sampling was performed to avoid overfitting. This warrants further study with a larger number of participants with TMDs. Furthermore, this research did not consider potential relationships or mediating effects among the independent variables. A subgroup analysis across age and sex would offer more insights into the major determinants of TMDs.

This study identified the etiologic factors that may be associated with the disease; efforts to eliminate the identified factors may help improve the prognosis. A recent study demonstrated the automated detection of temporomandibular joint arthritis from cone-beam computed tomography images using deep learning techniques^49^. When the machine learning methods from our study are combined with the image analysis algorithms, a personalized real-time diagnosis based on imaging data and demographic and biological records data may be possible. Possible risk factors may be identified for the purpose of determining a prognosis. This line of research is expected to break new ground for cutting-edge precision medicine concerning the diagnosis, prognosis, and treatment of TMDs.

## Conclusions

Artificial intelligence provides a decision-support system to predict TMDs and to analyze their determinants. Interventions regarding stress, socioeconomic status, and working conditions are needed for effective management of TMDs.

## Supplementary Information

Supplementary Tables.

## Data Availability

The data are available from the Korea Disease Control and Prevention Agency database from the following webpage: https://knhanes.kdca.go.kr/knhanes/sub03/sub03_02_05.do. The data are available to anyone with the appropriate qualifications.

## References

[1] Dworkin SF (2011). Temporomandibular disorder (TMD) pain-related disability found related to depression, nonspecific physical symptoms, and pain duration at 3 international sites. J. Evid. Based Dent. Pract..

[2] Ghurye S, McMillan R (2017). Orofacial pain—An update on diagnosis and management. Br. Dent. J..

[3] Okeson J, Jeffrey P (2019). Management of Temporomandibular Disorders and Occlusion-E Book.

[4] National Institute of Dental and Craniofacial Research. Facial Pain. Preprint at https://www.nidcr.nih.gov/research/data-statistics/facial-pain (2018).

[5] Schiffman E (2014). Diagnostic criteria for temporomandibular disorders (DC/TMD) for clinical and research applications: Recommendations of the International RDC/TMD Consortium Network and Orofacial Pain Special Interest Group. J. Oral Facial Pain Headache.

[6] National Institute of Dental and Craniofacial Research. Prevalence of TMJD and its signs and symptoms. Preprint at https://www.nidcr.nih.gov/research/data-statistics/facial-pain/prevalence (2018).

[7] Sessle BJ, Sessle BJ (2014). The societal, political, educational, scientific and clinical context of orofacial pain. Orofacial Pain. Recent Advances in Assessment, Management, and Understanding of Mechanisms.

[8] National Health Insurance Service (2019). Medical Aid Statistics.

[9] de Leeuw R, Klasser GD (2018). Orofacial Pain-Guidelines for Assessment, Diagnosis and Management.

[10] Gesch D, Bernhardt O, Alte D, Kocher T, John U, Hensel E (2004). Malocclusions and clinical signs or subjective symptoms of temporomandibular disorders (TMD) in adults. Results of the population-based Study of Health in Pomerania (SHIP). J. Orofac. Orthop..

[11] Jarabak JR (1956). An electromyographic analysis of muscular and temporomandibular joint disturbances due to imbalances in occlusion. Angle Orthod..

[12] Mundt T (2005). Gender differences in associations between occlusal support and signs of temporomandibular disorders: Results of the population-based Study of Health in Pomerania (SHIP). Int. J. Prosthodont..

[13] Lila-Krasniqi ZD, Shala KS, Pustina-Krasniqi T, Bicaj T, Dula LJ, Guguvčevski L (2015). Differences between centric relation and maximum intercuspation as possible cause for development of temporomandibular disorder analyzed with T-scan III. Eur. J. Dent..

[14] Schiffman EL, Fricton JR, Haley D (1992). The relationship of occlusion, parafunctional habits, and recent life events to mandibular dysfunction in a non-patient population. J. Oral Rehabil..

[15] Oikarinen KS, Raustia AM, Lathi J (1991). Signs and symptoms of TMJ dysfunction in patients with mandibular condyle fracture. J. Craniomandibular Pract..

[16] Huang GJ, LeResche L, Critchlow CW, Martin MD, Drangsholt MT (2002). Risk factors for diagnostic subgroups of painful temporomandibular disorders (TMD). J. Dent. Res..

[17] Salameh E, Alshaarani F, Abou Hamed H, Abou Nassar J (2015). Investigation of the relationship between psychosocial stress and temporomandibular disorder in adults by measuring salivary cortisol concentration: A case–control study. J. Indian Prosthodont. Soc..

[18] Ferreira DMAO, Costa YM, Bonjardim LR, Conti PCR (2021). Effects of acute mental stress on conditioned pain modulation in temporomandibular disorders patients and healthy individuals. J. Appl. Oral Sci..

[19] Wieckiewicz M (2014). Prevalence and correlation between TMD based on RDC/TMD diagnoses, oral parafunctions and psychoemotional stress in Polish university students. BioMed. Res. Int..

[20] Slade GD (2013). Signs and symptoms of first-onset TMD and sociodemographic predictors of its development: The OPPERA prospective cohort study. J. Pain.

[21] Nishiyama A, Kino K, Sugisaki M, Tsukagoshi K (2013). A survey of influence of work environment on temporomandibular disorders-related symptoms in Japan. Head Face Med..

[22] Ahlberg J (2004). Associations of perceived pain and painless TMD-related symptoms with alexithymia and depressive mood in media personnel with or without irregular shift work. Acta Odontol. Scand..

[23] Burris JL, Evans DR, Carlson CR (2010). Psychological correlates of medical comorbidities in patients with temporomandibular disorders. J. Am. Dent. Assoc..

[24] Skog C, Fjellner J, Ekberg E, Häggman-Henrikson B (2019). Tinnitus as a comorbidity to temporomandibular disorders—A systematic review. J. Oral Rehabil..

[25] Song HS (2018). Association between temporomandibular disorders, chronic diseases, and ophthalmologic and otolaryngologic disorders in Korean adults: A cross-sectional study. PLoS One.

[26] Kweon SKY (2014). Data resource profile: The Korea National Health and Nutrition Examination Survey (KNHANES). Int. J. Epidemiol..

[27] Van der Laan MJ (2006). Statistical inference for variable importance. Int. J. Biostat..

[28] Breiman L (2001). Random forests. Mach. Learn.

[29] Chisnoiu AM (2015). Factors involved in the etiology of temporomandibular disorders—A literature review. Clujul Med..

[30] Ohrbach R, Michelotti A (2018). The role of stress in the etiology of oral parafunction and myofascial pain. Oral Maxillofac. Surg. Clin. North Am..

[31] Ouanounou A, Goldberg M, Haas DA (2017). Pharmacotherapy in temporomandibular disorders: A review. J. Can. Dent. Assoc..

[32] Han W, Kwon SC, Lee YJ, Park C, Jang EC (2018). The associations between work-related factors and temporomandibular disorders among female full-time employees: Findings from the Fourth Korea National Health and Nutrition Examination Survey IV (2007–2009). Ann. Occup. Environ. Med..

[33] Sim SH, Ha M (2013). Association between psychological factors and temporomandibular disorders in Korean adults: The fourth Korean National Health and Nutritional Examination Survey (2009). J. Korean Soc. Dent. Hyg..

[34] Pratt CA (2017). A systematic review of obesity disparities research. Am. J. Prev. Med..

[35] Mohamed-Ali V, Pinkney JH, Coppack SW (1998). Adipose tissue as an endocrine and paracrine organ. Int. J. Obes. Relat. Metab. Disord..

[36] Okifuji A, Hare BD (2015). The association between chronic pain and obesity. J. Pain Res..

[37] Paulis WD, Silva S, Koes BW, van Middelkoop M (2014). Overweight and obesity are associated with musculoskeletal complaints as early as childhood: A systematic review. Obes. Rev..

[38] Smith SM, Sumar B, Dixon KA (2014). Musculoskeletal pain in overweight and obese children. Int. J. Obes. (Lond.).

[39] Berenbaum F, Eymard F, Houard X (2013). Osteoarthritis, inflammation and obesity. Curr. Opin. Rheumatol..

[40] Crowson CS, Matteson EL, Davis JM, Gabriel SE (2013). Contribution of obesity to the rise in incidence of rheumatoid arthritis. Arthritis Care Res..

[41] Dar L (2018). Are obesity and rheumatoid arthritis interrelated?. Int. J. Clin. Pract..

[42] Zhang Q, Wang Y (2004). Trends in the association between obesity and socioeconomic status in US adults: 1971 to 2000. Obes. Res..

[43] Centers for Disease Control and Prevention (2002). Prevalence of self-reported arthritis or chronic joint symptoms among adults: United States, 2001. MMWR Morb. Mortal. Wkly. Rep..

[44] Okoro CA, Hootman JM, Strine TW, Balluz LS, Mokdad AH (2004). Disability, arthritis, and body weight among adults 45 years and older. Obes. Res..

[45] Sandmark H, Hogstedt C, Lewold S, Vingard E (1999). Osteoarthrosis of the knee in men and women in association with overweight, smoking, and hormone. Ann. Rheum. Dis..

[46] Busija L, Hollingsworth B, Buchbinder R, Osborne RH (2007). Role of age, sex, and obesity in the higher prevalence of arthritis among lower socioeconomic groups: A population-based survey. Arthritis Care Res..

[47] Jordani PC (2017). Obesity as a risk factor for temporomandibular disorders. J. Oral Rehabil..

[48] LeResche L, Mancl LA, Drangsholt MT, Huang G, Von Korff M (2007). Predictors of onset of facial pain and temporomandibular disorders in early adolescence. Pain.

[49] Lee KS (2020). Automated detection of TMJ osteoarthritis based on artificial intelligence. J. Dent. Res..

