# Supplementary material for: Risk factor assessments of temporomandibular disorders via machine learning
Source: Sci Rep. 2021 Oct 5;11:19802. doi: 10.1038/s41598-021-98837-5 (PMC8492627; doi:10.1038/s41598-021-98837-5)
Supplement: Supplementary file 1 — Supplementary Tables. [file 41598_2021_98837_MOESM1_ESM.pdf]

Appendix Table 1. Descriptive statistics—categorical variables

| Variable | Count | Percentage |
|---|---|---|
| TMD (Self-Reported) | | |
| No | 4643 | 97.9 |
| Yes | 101 | 2.1 |
| TMD (Diagnosed by Doctor) | | |
| No | 4676 | 98.6 |
| Yes | 68 | 1.4 |
| Sex (Female) | | |
| No | 2479 | 52.3 |
| Yes | 2265 | 47.7 |
| Region: Metropolitan | | |
| No | 2735 | 57.7 |
| Yes | 2009 | 42.3 |
| Region: Rural | | |
| No | 3395 | 71.6 |
| Yes | 1349 | 28.4 |
| Residence Type: Apartment | | |
| No | 2912 | 61.4 |
| Yes | 1832 | 38.6 |
| Household Income - Subjective | | |
| Q1 | 1139 | 24.0 |
| Q2 | 1216 | 25.6 |
| Q3 | 1195 | 25.2 |
| Q4 | 1194 | 25.2 |
| Education | | |
| Elementary school and below | 1034 | 21.8 |
| Junior High | 543 | 11.4 |
| Senior High | 1755 | 37.0 |
| College and above | 1412 | 29.8 |
| Occupation | | |
| Unemployed | 435 | 9.2 |
| Agriculture | 632 | 13.3 |
| Manufacturing | 693 | 14.6 |
| Service | 2984 | 62.9 |
| Marital status | | |
| Married | 3450 | 72.7 |
| Separated | 63 | 1.3 |
| Widowed | 217 | 4.6 |
| Divorced | 169 | 3.6 |
| Unmarried | 845 | 17.8 |
| Health Insurance | | |
| Medicare | 4654 | 98.1 |
| Medicaid | 90 | 1.9 |
| Stress | | |
| No | 3268 | 68.9 |

| | | |
|---|---|---|
| Yes | 1476 | 31.1 |
| Suicidal Ideation | | |
| No | 4038 | 85.1 |
| Yes | 706 | 14.9 |
| Working environment - Hygiene | | |
| Very Poor | 151 | 3.2 |
| Poor | 839 | 17.7 |
| Good | 3023 | 63.7 |
| Very Good | 731 | 15.4 |
| Working environment - Risk | | |
| Very Small | 1638 | 34.5 |
| Small | 1769 | 37.3 |
| Big | 1176 | 24.8 |
| Very Big | 161 | 3.4 |
| Working environment - Workload | | |
| Very Small | 674 | 14.2 |
| Small | 2170 | 45.7 |
| Big | 1633 | 34.4 |
| Very Big | 267 | 5.6 |
| Working environment - Control | | |
| Very Poor | 236 | 5.0 |
| Poor | 1035 | 21.8 |
| Good | 2827 | 59.6 |
| Very Good | 646 | 13.6 |
| Working environment - Respect | | |
| Very Poor | 52 | 1.1 |
| Poor | 396 | 8.3 |
| Good | 3825 | 80.6 |
| Very Good | 471 | 9.9 |
| Smoking status | | |
| No | 2473 | 52.1 |
| Yes | 2271 | 47.9 |
| Subjective Health | | |
| Very Good | 245 | 5.2 |
| Good | 1892 | 39.9 |
| Middle | 1640 | 34.6 |
| Poor | 867 | 18.3 |
| Very Poor | 100 | 2.1 |
| Obesity - Subjective | | |
| Very Thin | 164 | 3.5 |
| Thin | 620 | 13.1 |
| Middle | 1916 | 40.4 |
| Obese | 1635 | 34.5 |
| Very Obese | 409 | 8.6 |
| Drinking | | |
| No | 486 | 10.2 |
| Yes | 4258 | 89.8 |

| | | |
|---|---|---|
| Hypertensive Disorders | | |
| No | 4017 | 84.7 |
| Yes | 727 | 15.3 |
| Rheumatoid Arthritis | | |
| No | 4317 | 91.0 |
| Yes | 427 | 9.0 |
| Osteoarthritis | | |
| No | 4369 | 92.1 |
| Yes | 375 | 7.9 |
| Osteoporosis | | |
| No | 4534 | 95.6 |
| Yes | 210 | 4.4 |
| Lumbago | | |
| No | 4094 | 86.3 |
| Yes | 650 | 13.7 |
| Sinusitis | | |
| No | 4546 | 95.8 |
| Yes | 198 | 4.2 |
| Allergic Rhinitis | | |
| No | 4249 | 89.6 |
| Yes | 495 | 10.4 |
| Mental Depression | | |
| No | 4625 | 97.5 |
| Yes | 119 | 2.5 |
| Atopic Dermatitis | | |
| No | 4609 | 97.2 |
| Yes | 135 | 2.8 |
| Diabetes Miletus | | |
| No | 4495 | 94.8 |
| Yes | 249 | 5.2 |
| Thyroid Disorders | | |
| No | 4628 | 97.6 |
| Yes | 116 | 2.4 |
| Otitis Media | | |
| No | 4596 | 96.9 |
| Yes | 148 | 3.1 |
| Gastric/Duodenal Ulcer | | |
| No | 4464 | 94.1 |
| Yes | 280 | 5.9 |

Appendix Table 2. Logistic regression results: odds ratios, (** P < 0.05, * P < 0.10)

| **Variable** | **Odds Ratio** | | | |
|---|---|---|---|---|
| | TMD (Self-Reported) | | TMD (Diagnosed by Doctor) | |
| Region (Metropolitan) | | | | |
| No | | | | |
| Yes | 0.8768 | | 0.5994 | |
| Region (Rural) | | | | |
| No | | | | |
| Yes | 0.5937 | | 0.4556 | |
| Residence Type (Apartment) | | | | |
| No | | | | |
| Yes | 1.0156 | | 0.8129 | |
| Sex (Female) | | | | |
| No | | | | |
| Yes | 1.4331 | | 1.6001 | |
| Household Income - Subjective | | | | |
| Q1 | | | | |
| Q2 | 0.8553 | | 0.5181 | |
| Q3 | 0.4914 | | 0.9861 | |
| Q4 | 0.7129 | | 0.7509 | |
| Education | | | | |
| Elementary, Below | | | | |
| Junior High | 0.1853 | | 0.1916 | |
| Senior High | 0.8176 | | 0.8750 | |
| College, Above | 0.8318 | | 0.6471 | |
| Occupation | | | | |
| Unemployed | | | | |
| Agriculture | 1.5271 | | 0.7145 | |
| Manufacturing | 0.6713 | | 0.3899 | |
| Service | 0.7029 | | 0.7169 | |
| Marriage | | | | |
| Married | | | | |
| Separated | <0.0001 | ** | <0.0001 | ** |
| Widowed | <0.0001 | ** | <0.0001 | ** |
| Divorced | 1.5424 | | 0.6979 | |
| Unmarried | 0.7760 | | 1.1188 | |
| Health Insurance | | | | |
| Medicare | | | | |
| Medicaid | 0.8935 | | <0.0001 | ** |
| Working Condition - Hygiene | | | | |
| Very Poor | | | | |
| Poor | 2.3751 | | 1.4144 | |

| | | | | |
|---|---|---|---|---|
| Good | 2.0309 | | 2.1618 | |
| Very Good | 2.0930 | | 2.1871 | |
| Working Condition - Risk | | | | |
| Very Small | | | | |
| Small | 0.8423 | | 0.9052 | |
| Big | 0.8673 | | 1.1848 | |
| Very Big | 0.7979 | | 0.6837 | |
| Working Condition - Workload | | | | |
| Very Small | | | | |
| Small | 2.5790 | * | 1.4827 | |
| Big | 2.4328 | * | 1.0046 | |
| Very Big | 1.9097 | | 0.2952 | |
| Working Condition - Control | | | | |
| Very Poor | | | | |
| Poor | 0.7320 | | 1.4098 | |
| Good | 1.1890 | | 1.0284 | |
| Very Good | 2.1730 | | 0.8682 | |
| Working Condition - Respect | | | | |
| Very Poor | | | | |
| Poor | 0.4805 | | 91863.6100 | ** |
| Good | 0.5119 | | 80734.4800 | ** |
| Very Good | 0.4652 | | 154111.2000 | ** |
| Obesity - Subjective | | | | |
| Very Thin | | | | |
| Thin | 1.2325 | | 0.9955 | |
| Middle | 0.5795 | | 0.9627 | |
| Obese | 0.4503 | | 0.7050 | |
| Very Obese | 0.2311 | | 0.7879 | |
| Drinking | | | | |
| No | | | | |
| Yes | 0.9198 | | 0.7063 | |
| Stress | | | | |
| No | | | | |
| Yes | 0.6401 | | 1.9960 | ** |
| Suicidal Ideation | | | | |
| No | | | | |
| Yes | 2.4388 | ** | 2.2270 | ** |
| Smoking status | | | | |
| No | | | | |
| Yes | 1.0162 | | 1.2637 | |
| Subjective Health | | | | |
| Very Good | | | | |
| Good | 1.1045 | | 1.8899 | |
| Middle | 2.4454 | | 3.1048 | |

| | | | | |
|---|---|---|---|---|
| Poor | 1.9512 | | 2.0326 | |
| Very Poor | 2.4530 | | 1.2925 | |
| Hypertensive Disorders | | | | |
| No | | | | |
| Yes | 0.6078 | | 0.4607 | |
| Rheumatoid Arthritis | | | | |
| No | | | | |
| Yes | 2.9455 | | 1.6877 | |
| Osteoarthritis | | | | |
| No | | | | |
| Yes | 0.1132 | | 0.0000 | |
| Osteoporosis | | | | |
| No | | | | |
| Yes | 1.6133 | | 0.9295 | |
| Lumbago | | | | |
| No | | | | |
| Yes | 1.3984 | | 2.4876 | ** |
| Sinusitis | | | | |
| No | | | | |
| Yes | 1.2305 | | 1.3601 | |
| Allergic Rhinitis | | | | |
| No | | | | |
| Yes | 1.8412 | * | 1.7513 | |
| Mental Depression | | | | |
| No | | | | |
| Yes | 1.6355 | | 3.5054 | ** |
| Atopic Dermatitis | | | | |
| No | | | | |
| Yes | 0.8443 | | 0.4911 | |
| Diabetes Miletus | | | | |
| No | | | | |
| Yes | 1.4099 | | 0.6900 | |
| Thyroid Disorders | | | | |
| No | | | | |
| Yes | 1.1854 | | 3.2357 | * |
| Otitis Media | | | | |
| No | | | | |
| Yes | 1.2011 | | 2.0550 | |
| Gastric/Duodenal Ulcer | | | | |
| No | | | | |
| Yes | 1.5726 | | 1.5865 | |
| Age | 0.9298 | ** | 0.9620 | * |
| Household Income - Monthly | 1.0001 | | 1.0008 | |
| Body Mass Index | 1.1031 | * | 1.1103 | |

| | | |
|---|---|---|
| Sleep - Daily | 0.8934 | 0.8288 |
